# Comparative analysis of apicoplast genomes of *Babesia* infective to small ruminants in China

**DOI:** 10.1186/s13071-019-3581-x

**Published:** 2019-06-24

**Authors:** Xiaoxing Wang, Jinming Wang, Junlong Liu, Aihong Liu, Xin He, Jianlin Xu, Zhi Li, Shuaiyang Zhao, Youquan Li, Hong Yin, Jianxun Luo, Guiquan Guan

**Affiliations:** 1 State Key Laboratory of Veterinary Etiological Biology, Key Laboratory of Veterinary Parasitology of Gansu Province, Lanzhou Veterinary Research Institute, Chinese Academy of Agricultural Science, Xujiaping 1, Lanzhou, 730046 Gansu People’s Republic of China; 2 Jiangsu Co-Innovation Center for the Prevention and Control of Important Animal Infectious Disease and Zoonose, Yangzhou University, Yangzhou, 225009 People’s Republic of China

**Keywords:** *Babesia motasi*, *Babesia* sp., Apicoplast genome, Assembly and annotation, Comparative analysis

## Abstract

**Background:**

Babesiosis is an economically important disease caused by tick-borne apicomplexan protists of the genus *Babesia*. Most apicomplexan parasites, including *Babesia*, have a plastid-derived organelle termed an apicoplast, which is involved in critical metabolic pathways such as fatty acid, iron-sulphur, haem and isoprenoid biosynthesis. Apicoplast genomic data can provide significant information for understanding and exploring the biological features, taxonomic and evolutionary relationships of apicomplexan parasites, and identify targets for anti-parasitic drugs. However, there are limited data on the apicoplast genomes of *Babesia* species infective to small ruminants.

**Methods:**

PCR primers were designed based on the previously reported apicoplast genome sequences of *Babesia motasi* Lintan and *Babesia* sp. Xinjiang using Illumina technology. The overlapped apicoplast genomic fragments of six ovine *Babesia* isolates were amplified and sequenced using the Sanger dideoxy chain-termination method. The full-length sequences of the apicoplast genomes were assembled and annotated using bioinformatics software. The gene contents and order of apicoplast genomes obtained in this study were defined and compared with those of other apicomplexan parasites. Phylogenetic trees were constructed on the concatenated amino acid sequences of 13 gene products using MEGA v.6.06.

**Results:**

The results showed that the six ovine *Babesia* apicoplast genomes consisted of circular DNA. The genome sizes were 29,916–30,846 bp with 78.7–81.0% A + T content, 29–31 open reading frames (ORF) and 23–24 transport RNAs. The ORFs encoded four DNA-directed RNA polymerase subunits (*rpo*B, *rpo*Cl, *rpo*C2a and *rpo*C2b), 13 ribosomal proteins, one elongation factor TU (*tuf*A), two ATP-dependent Clp proteases (*Clp*C) and 7–11 hypothetical proteins. *Babesia* sp. has three more genes than *Babesia motasi* (*rpl*5, *rps*8 and *rpo*B). Phylogenetic analysis showed that *Babesia* sp. is located in a separate clade. *Babesia motasi* Lintan/Tianzhu and *B. motasi* Ningxian/Hebei were divided into two subclades.

**Conclusions:**

To our knowledge, this study is the first to elucidate the whole apicoplast genomic structural features of six *Babesia* isolates infective to small ruminants in China using Sanger sequencing. The data provide useful information confirming the taxonomic relationships of these parasites and identifying targets for anti-apicomplexan parasite drugs.

**Electronic supplementary material:**

The online version of this article (10.1186/s13071-019-3581-x) contains supplementary material, which is available to authorized users.

## Background

Most apicomplexan protists cause important diseases of humans and other animals, including malaria (*Plasmodium* spp.), toxoplasmosis (*Toxoplasma gondii*), cryptosporidiosis (*Cryptosporidium* spp.), cyclosporiasis (*Cyclospora cayetanensis*), coccidiosis (*Eimeria* spp.), babesiosis (*Babesia* spp.), theileriosis (*Theileria* spp.) and neosporosis (*Neospora caninum*) [1]. These parasites, with the exception of *Cryptosporidium* spp. [2], possess a unique, essential, vestigial plastid known as the apicoplast. The discovery of the photosynthetic apicomplexan *Chromera* (chromalveolate hypothesis), the structural characteristics of the apicoplast/plastid genome and phylogenetic analysis of the glyceraldehyde-3-phosphate dehydrogenase and cytochrome *c* oxidase subunit 2 genes have demonstrated that the apicoplast has evolved through secondary endosymbiosis of a red alga [3–6]. The apicoplast is also involved in critical metabolic pathways such as fatty acid, iron-sulphur, haem and isoprenoid biosynthesis. Previous studies have shown that *Plasmodium falciparum*, *Babesia bovis* and *Babesia bigemina* cannot grow with the antibiotic fosmidomycin that causes loss of the apicoplast [7, 8]. Interestingly, isopentenyl pyrophosphate supplementation completely reverses death following treatment with fosmidomycin [8]. The apicoplast and some of these metabolic pathways are vital for parasite survival, thus making the apicoplast an attractive target for anti-parasitic drugs.

*Babesia* spp. are tick-transmitted haemoprotozoa with a worldwide distribution that have been reported to affect domestic animals, wildlife, companion animals and humans [9]. These pathogens cause fever, anaemia, haemoglobinuria and jaundice in acute infections, while chronic infection is asymptomatic. Babesiosis of cattle and small ruminants also has great economic importance due to reduced meat and milk production, and costs associated with treatment, prevention and the disposing of carcasses. The main causative agents of babesiosis in small ruminants are *Babesia ovis* and *Babesia motasi*, which are transmitted by *Rhipicephalus* spp. and *Haemaphysalis* spp and are distributed in Asia, Africa, South America, Europe and the Far East [9–11]. To date, several ovine *Babesia* species or geographical isolates, including *Babesia* sp. Xinjiang (BspXJ), *Babesia* sp. Dunhuang (BspDH), *B. motasi* Lintan (BmLT), *B. motasi* Tianzhu (BmTZ), *B. motasi* Hebei (BmHB) and *B. motasi* Ningxian (BmNX), have been isolated from small ruminants in China. They have significant differences in vector specificity, serology, virulence and pathogenicity. Importantly, BspXJ and BspDH are transmitted by *Hyalomma anatolicum* and mainly cause subclinical infections, whereas BmLT, BmTZ, BmHB and BmNX are transmitted by *Haemaphysalis* spp. and cause mild to severe clinical signs [12–20]. Seroepidemiological surveys by enzyme-linked immunosorbent assay (ELISA) have shown that the prevalence of *Babesia* sp. and *B. motasi* are 30.4–31.7% and 36.0–43.5%, respectively [19, 21–23]. These data indicate that ovine *Babesia* spp. are widespread in China.

Thus far, sequencing and annotation of the apicoplast genomes have been performed for *Plasmodium* spp., *T. gondii*, *Leucocytozoon caulleryi*, *Cyclospora cayetanensis*, *Theileria parva*, *Eimeria tenella*, *B. bovis*, *Babesia microti*, *Babesia orientalis*, BspXJ and BmLT. These studies have revealed that apicoplast genomes lack the genes for metabolic function and regulatory proteins, but are present SNPs and repeats [24–37]. Apicoplast genomic data have been used to understand and explore the biological features of the pathogens, and the taxonomy and evolutionary relationships among apicomplexan parasites [33, 36]. However, there is limited information on the apicoplast genomes of *Babesia* species infective to small ruminants in China. Although the apicoplast genomes of BspXJ and BmLT have been sequenced and characterized using Illumina technology directly from genomic DNA, they were not verified using PCR amplification and sequencing [36].

In the present study, we employed a Sanger dideoxy chain-termination method to sequence the apicoplast genomes of BspXJ, BspDH, BmLT, BmTZ, BmHB and BmNX, and used bioinformatics software to conduct the assembly and annotation of full sequences of these isolates to verify the published sequences of BspXJ and BmLT sequenced using Illumina technology. Phylogenetic trees were constructed using the apicoplast genomic data to determine the taxonomic relationships among these geographical isolates of *Babesia* infective to sheep and goats in China. The study provides essential data for clarifying the classification of the *Babesia* species infective to small ruminants and lays the foundation for performing research on metabolic pathways and identifying diagnostic markers and drug targets for *Babesia* infections.

## Methods

### Parasites and isolation of genomic DNA

BspXJ, BspDH, BmLT, BmTZ, BmHB and BmNX were isolated from splenectomised sheep that were infected or infested with field-collected sheep blood or ticks from Xinjiang, Hebei and Gansu (Lintan, Tianzhu, Ningxian and Dunhuang counties) provinces during the period 2000–2010. The purified merozoites of BspXJ, BspDH, BmLT, BmTZ, BmHB and BmNX were provided by the vectors and vector-borne diseases laboratory in Lanzhou Veterinary Research Institute, China [38]. Genomic DNA was extracted from the merozoites using a QIAamp DNA Blood Mini Kit (Qiagen, Hilden, Germany), according to the manufacturer’s instructions. The DNA concentration and quality were measured using the 260/280 nm absorbance ratio on a NanoDrop spectrophotometer (Thermo Fisher Scientific, Waltham, MA, USA). DNA was stored at − 20 °C until PCR amplification.

### Amplification and sequencing of apicoplast genomes

The PCR primers were designed on the basis of the reported apicoplast genomic sequences of BspXJ (KX881914) and BmLT (KX881915), sequenced using Illumina technology [36]. The primer details are shown in Additional file 1: Table S1. Overlapped fragments covering whole apicoplast genomes were amplified from the genomic DNA of BspXJ, BspDH, BmLT, BmTZ, BmHB and BmNX. The PCR products were directly sequenced using a BigDye Terminator v.3.1 cycle sequencing kit (Applied Biosystems, Foster City, CA, USA) on an ABI 3730 DNA analyser (Applied Biosystems) or cloned into pBluescript II SK(+) using a Clone Express® II One Step Cloning Kit (Vazyme, Nanjing, China) for subsequent sequencing.

### Assembly and annotation of the apicoplast genomes

The CLC Genomics Workbench v.7.5.1 (Qiagen, Redwood City, CA, USA) was used to assemble the apicoplast genomes according to the user manual. Genome annotation was performed using the software Artemis [39] and BLAST (http://blast.ncbi.nlm.nih.gov/Blast.cgi). The use of these two methods guarantees the robustness of the annotation. The E-value of BLAST is 0.0 and we were blasting against a non-redundant protein sequence (nr) database. The tRNA genes were identified using tRNAscan-SE v.2.0 (http://lowelab.ucsc.edu/tRNAscan-SE/) with the default search mode and other mitochondrial sequence sources [40]. Genetic maps were obtained using the online software CGView (http://stothard.afns.ualberta.ca/cgview_server/) [41]. Mauve (http://gel.ahabs.wisc.edu/mauve) was used to generate the genome comparisons [42]. The nucleotide sequences and annotation information reported in this article were submitted to the GenBank database under the accession numbers MH992224-MH992229.

### Phylogenetic analysis

The 18 apicoplast genomic sequences, six from the ovine *Babesia* isolates from this study and 12 from apicomplexan parasites obtained from GenBank [*Babesia* sp. Xinjiang (BspXJ), *B. motasi* Lintan (BmLT), *B. bovis*, *B. orientalis*, *B. microti*, *Theileria parva*, *Plasmodium falciparum*, *Cyclospora cayetanensis*, *P. chabaudi chabaudi*, *Toxoplasma gondii*, *Eimeria tenella* and *Leucocytozoon caulleryi*], and one chloroplast genomic sequence of *Chromera velie* (GenBank: HM222967), were used in the phylogenetic analysis (Table 1). On the basis of the annotation information, 13 encoding genes (*rpl*2, *rpl*4, *rpl*6, *rpl*14, *rpl*16, *rps*2, *rps*3, *rps*4, *rps*7, *rps*11, *rps*12, *tuf*A and *rop*B) of each genomic sequence were used to deduce their amino acid sequences. The concatenated sequences of 4683 amino acid residues from each species were put into multi-alignment using Clustal W with further manual verification. Subsequently, MEGA v.6.06 (http://www.megasoftware.net/) software was applied to conduct phylogenetic analysis. A bootstrap phylogenetic tree demonstrating the relationship of BspXJ, BspDH, BmLT, BmTZ, BmNX and BmHB to other apicomplexan parasites was created by the maximum likelihood (ML) method or neighbour-joining (NJ) method, using a distance matrix corrected for nucleotide substitutions based on the JTT with Freqs model. In addition, phylogeny of the whole apicoplast nucleotide sequence was constructed by the ML method based on the Kimura 2-parameter model. A bootstrap analysis was used to assess the robustness of the clusters using 1000 replicates.

**Table 1:** Comparison of apicoplast genome sequences from 16 apicomplexan parasites

Species	Host	GenBank ID	Size (bp)	A+T (%)	Total no. of genes	Protein-encoding genes	rRNA	tRNA	Reference
*Babesia* sp. Xinjiang (BspXJ-Sanger)^a1^	Sheep	MH992224	30,758	81.0	55	30	2	23	This study
*Babesia* sp. Dunhuang (BspDH)	Sheep	MH992225	30,771	81.0	55	30	2	23	This study
*Babesia motasi* Lintan (BmLT-Sanger)^b1^	Sheep	MH992226	30,846	78.7	57	31	2	24	This study
*Babesia motasi* Tianzhu (BmTZ)	Sheep	MH992227	30,846	78.7	57	31	2	24	This study
*Babesia motasi* Ningxian (BmNX)	Sheep	MH992228	29,916	79.1	54	29	2	23	This study
*Babesia motasi* Hebei (BmHB)	Sheep	MH992229	29,921	79.1	54	29	2	23	This study
*Babesia* sp. Xinjiang (BspXJ-Illumina)^a2^	Sheep	KX881914	30,729	81.0	57	30	2	25	[36]
*Babesia motasi* Lintan (BmLT-Illumina)^b2^	Sheep	KX881915	30,738	78.7	59	32	2	25	[36]
*Babesia bovis*	Cattle	NC011395	35,107	78.0	58	32^c^	2	24	[28]
*Babesia orientalis*	Water buffalo	KT428643	33,200	79.0	64	38	2	24	[24]
*Babesia microti*	Mouse	LK028575	28,657	85.9	54	28^c^	2	24	[25]
*Theileria parva*	Cattle	NC007758	39,579	80.5	70	44^c^	2	24	[37]
*Plasmodium falciparum*	Human	LN999985	34,250	85.8	68	30	4	34	Unpublished
*Cyclospora cayetanensis*	Human	KP866208	34,155	78.0	65	28	4	33	[44]
*Plasmodium chabaudi chabaudi*	Mouse	HF563595	29,623	86.3	59	30	2^d^	27^e^	[35]
*Toxoplasma gondii*	Mouse	U87145	34,996	78.6	65	28	4	33	Unpublished
*Eimeria tenella*	Chicken	AY217738	34,750	79.4	65	28	4	33	[29]
*Leucocytozoon caulleryi*	Chicken	AP013071	34,779	85.1	67	29	4	34	[33]

^a^a1 and a2 are the same sample, sequenced using the Sanger and Illumina method, respectively

^b^b1 and b2 are the same sample, sequenced using the Sanger and Illumina method, respectively

^c^Two rpoC2 genes were counted as one gene

^d^One large subunit rRNA has a large deletion and was thus removed from the gene count

^e^One tRNA-R(acg) was removed from the gene count, as it does not have a predicted anticodon

## Results

### Sequence analysis of the apicoplast genomes of ovine *Babesia*

Sequencing and assembly revealed that the apicoplast genomes of the six ovine *Babesia* isolates were formed of circular DNA, ranging from 29,916 to 30,846 bp in length with a high A + T content of 78.7–81.0% (Table 1). Bioinformatics analysis indicated that the circular DNA contained a small subunit and a large subunit ribosomal RNA (*SSU* and *LSU*), 23–24 transfer ribonucleic acids (tRNAs) and five to six ribosomal protein large subunits (*rpl*), eight to nine ribosomal protein small subunits (*rps*), four to five subunits of DNA-directed RNA polymerase (*rpo*), two ATP-dependent Clp proteases (*clp*C1, *clp*C2), one elongation factor TU (*tuf*A), and seven to eleven hypothetical protein genes (*hyp*) (Table 2).

**Table 2:** Gene contents of the apicoplast genomes of six ovine *Babesia* isolates

Class	*Babesia* sp. Xinjiang	*Babesia* sp. Dunhuang	*Babesia motasi* Lintan	*Babesia motasi* Tianzhu	*Babesia motasi* Ningxian	*Babesia motasi* Hebei
Ribosomal RNA	*LSU*, *SSU*	*LSU*, *SSU*	*LSU*, *SSU*	*LSU*, *SSU*	*LSU*, *SSU*	*LSU*, *SSU*
Transfer RNA	Thr^TGT^, Gly^TCC^, Met^CAT^, His^GTG^, Ser^GCT^, Asp^GTC^, Glu^TTC^, Tyr^GTA^, Cys^GCA^, Lys^TTT^, Asn^GTT^, Pro^TGG^, Ser^TGA^, Gln^TTG^, Trp^CCA^, Arg^TCT^, Met^CAT^, Phe^GAA^, Leu^TAG^, Val^TAC^, Ala^TGC^, Ile^GAT^, Arg^ACG^	Thr^TGT^, Gly^TCC^, Met^CAT^, His^GTG^, Ser^GCT^, Asp^GTC^, Glu^TTC^, Tyr^GTA^, Cys^GCA^, Lys^TTT^, Asn^GTT^, Pro^TGG^, Ser^TGA^, Gln^TTG^, Trp^CCA^, Arg^TCT^, Met^CAT^, Phe^GAA^, Leu^TAG^, Val^TAC^, Ala^TGC^, Ile^GAT^, Arg^ACG^	Thr^TGT^, Gly^TCC^, Met^CAT^, His^GTG^, Ser^GCT^, **Lys**^**TTT**^, Asp^GTC^, Glu^TTC^, Tyr^GTA^, Cys^GCA^, Lys^TTT^, Asn^GTT^, Pro^TGG^, Ser^TGA^, Gln^TTG^, Trp^CCA^, Arg^TCT^, Met^CAT^, Phe^GAA^, Leu^TAG^, Val^TAC^, Ala^TGC^, Ile^GAT^, Arg^ACG^	Thr^TGT^, Gly^TCC^, Met^CAT^, His^GTG^, Ser^GCT^, **Lys**^**TTT**^, Asp^GTC^, Glu^TTC^, Tyr^GTA^, Cys^GCA^, Lys^TTT^, Asn^GTT^, Pro^TGG^, Ser^TGA^, Gln^TTG^, Trp^CCA^, Arg^TCT^, Met^CAT^, Phe^GAA^, Leu^TAG^, Val^TAC^, Ala^TGC^, Ile^GAT^, Arg^ACG^	Thr^TGT^, Gly^TCC^, Met^CAT^, His^GTG^, Ser^GCT^, Asp^GTC^, Glu^TTC^, Tyr^GTA^, Cys^GCA^, Lys^TTT^, Asn^GTT^, Pro^TGG^, Ser^TGA^, Gln^TTG^, Trp^CCA^, Arg^TCT^, Met^CAT^, Phe^GAA^, Leu^TAG^, Val^TAC^, Ala^TGC^, Ile^GAT^, Arg^ACG^	Thr^TGT^, Gly^TCC^, Met^CAT^, His^GTG^, Ser^GCT^, Asp^GTC^, Glu^TTC^, Tyr^GTA^, Cys^GCA^, Lys^TTT^, Asn^GTT^, Pro^TGG^, Ser^TGA^, Gln^TTG^, Trp^CCA^, Arg^TCT^, Met^CAT^, Phe^GAA^, Leu^TAG^, **Undet**^**NNN**^, Ala^TGC^, Ile^GAT^, Arg^ACG^
Ribosomal proteins	*rpl*2, *rpl*4, ***rpl*5**, *rpl*6, *rpl*14, *rpl*16; *rps*2, *rps*3, *rps*4, *rps*5, *rps*7, ***rps*8**, *rps*11, *rps*12, *rps*19,	*rpl*2, *rpl*4, ***rpl*5**, *rpl*6, *rpl*14, *rpl*16; *rps*2, *rps*3, *rps*4, *rps*5, *rps*7, ***rps*8**, *rps*11, *rps*12, *rps*19,	*rpl*2, *rpl*4, *rpl*6, *rpl*14, *rpl*16; *rps*2, *rps*3, *rps*4, *rps*5, *rps*7, *rps*11, *rps*12, *rps*19	*rpl*2, *rpl*4, *rpl*6, *rpl*14, *rpl*16; *rps*2, *rps*3, *rps*4, *rps*5, *rps*7, *rps*11, *rps*12, *rps*19	*rpl*2, *rpl*4, *rpl*6, *rpl*14, *rpl*16; *rps*2, *rps*3, *rps*4, *rps*5, *rps*7, *rps*11, *rps*12, *rps*19	*rpl*2, *rpl*4, *rpl*6, *rpl*14, *rpl*16; *rps*2, *rps*3, *rps*4, *rps*5, *rps*7, *rps*11, *rps*12, *rps*19
RNA polymerase	*rpo*B1, ***rpo*B2**, *rpo*C1, *rpo*C2a, *rpo*C2b	*rpo*B1, ***rpo*B2**, *rpo*C1, *rpo*C2a, *rpo*C2b	*rpo*B, *rpo*C1, *rpo*C2a, *rpo*C2b	*rpo*B, *rpo*C1, *rpo*C2a, *rpo*C2b	*rpo*B, *rpo*C1, *rpo*C2a, *rpo*C2b	*rpo*B, *rpo*C1, *rpo*C2a, *rpo*C2b
Other proteins	*clp*C1, *clp*C2, *tuf*A	*clp*C1, *clp*C2, *tuf*A	*clp*C1, *clp*C2, *tuf*A	*clp*C1, *clp*C2, *tuf*A	*clp*C1, *clp*C2, *tuf*A	*clp*C1, *clp*C2, *tuf*A
Unassigned ORFs	**7** ORFs (*hyp*1-7)	**7** ORFs (*hyp*1-7)	**11** ORFs (*hyp*1-11)	**11** ORFs (*hyp*1-11)	**9** ORFs (*hyp*1-9)	**9** ORFs (*hyp*1-9)

Note: The bold indicates that *Babesia* sp. are different from *Babesia motasi* in Gene contents of the apicoplast genomes

All coding genes were transcribed in the same orientation (Additional file 2: Figure S1; Additional file 3: Figure S2; Additional file 4: Figure S3; Additional file 5: Figure S4; Additional file 6: Figure S5; Additional file 7: Figure S6). In total, 7094, 7220, 7384, 7436, 7163 and 7212 amino acids were encoded in the apicoplast genomes of the six ovine *Babesia* species. All the protein-encoding genes had ATG as a translation start codon. Most of the apicoplast protein-encoding genes had TAA as a translation stop codon, followed by TGA and TAG (Table 3). The alignment of apicoplast genomes of ovine *Babesia* isolates indicated that the identities of BspXJ/DH, BmLT/TZ and BmNX/HB were 99.8, 99.9 and 99.9%, respectively. BmLT/TZ and BmNX/HB had 70.9 and 71.6–71.7% identity, respectively, to BspXJ/DH, and that between BmLT/TZ and BmNX/HB was 86.9–87.0%. Based on the nucleotide sequence analysis of the whole apicoplast, there were multiple base differences among BspXJ/DH, BmLT/TZ and BmNX/HB (Additional file 8: Table S2).

**Table 3:** Initiation codons and termination codons used in encoding genes of the six ovine *Babesia* apicoplast genomes

Species	Total no. of protein-encoding genes	Initiation codon				Termination codon		
		ATG	ATA	ATT	ATC	TAA	TGA	TAG
*Babesia* sp. Xinjiang^a1^ (BspXJ-Sanger, MH992224)	30	30	0	0	0	26	4	0
*Babesia* sp. Xinjiang^a2^ (BspXJ-Illumina, KX881914)	30	24	2	3	1	30	0	0
*Babesia* sp. Dunhuang (BspDH, MH992225)	30	30	0	0	0	26	4	0
*Babesia motasi* Lintan^b1^ (BmLT-Sanger, MH992226)	31	31	0	0	0	29	1	1
*Babesia motasi* Lintan^b2^ (BmLT-Illumina, KX881915)	32	30	2	0	0	30	0	2
*Babesia motasi* Tianzhu (BmTZ, MH992227)	31	31	0	0	0	29	1	1
*Babesia motasi* Ningxian (BmNX, MH992228)	29	29	0	0	0	27	1	1
*Babesia motasi* Hebei (BmHB, MH992229)	29	29	0	0	0	27	1	1

^a^ a1 and a2 are the same sample, sequenced using the Sanger and Illumina method, respectively

^b^ b1 and b2 are the same sample, sequenced using the Sanger and Illumina method, respectively

### Alignment of apicoplast genomes with those of other apicomplexan parasites

**Fig. 1:**
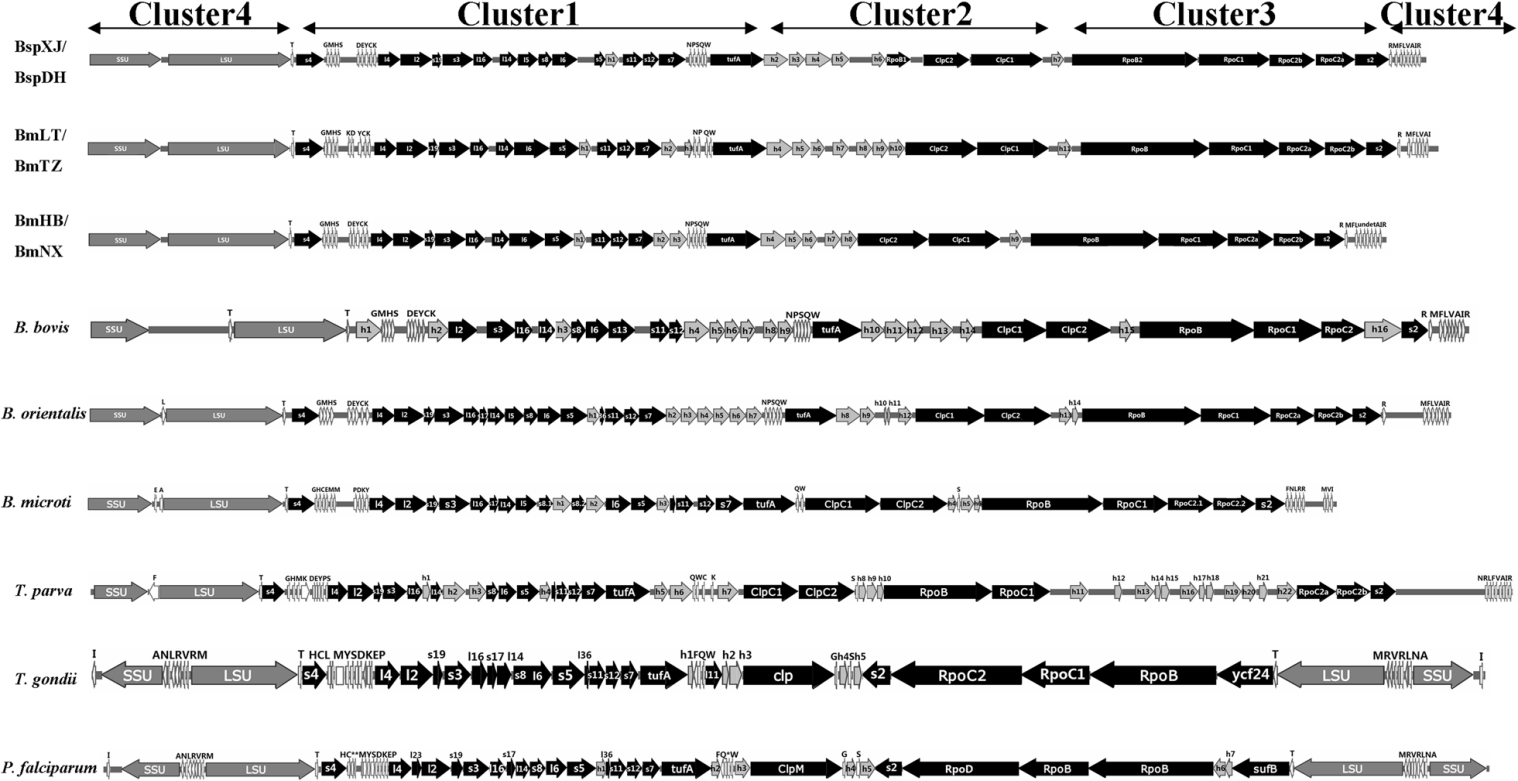
Comparison of the apicoplast genomes of six ovine *Babesia* isolates and other apicomplexan parasites. The circular apicoplast genomes are displayed in a linear format, beginning with the small subunit rRNA genes. It was performed using SnapGene software and Adobe Photoshop. The different shades of gray represent different gene types. In detail, white represents tRNA, 25% grey represents hypothetical protein, 50% grey represents *SSU* and *LSU*, and black represents already annotated functional protein

Four gene clusters were found in the BspXJ/DH, BmLT/TZ and BmHB/NX apicoplast genomes. They were in synteny with the same gene clusters of other apicomplexan parasites (Additional file 9: Figure S7). Gene cluster 1 included those encoding ribosomal proteins and *tuf*A genes (Fig. 1). Similar to the gene organization of *B. bovis*, *B. orientalis*, *B. microti*, *T. parva*, *T. gondii*, *E. tenella* and *C. cayetanensis* apicoplast genomes, the six ovine *Babesia* isolates lacked the *rpl*23 gene, but it was present in *P. chabaudi chabaudi* and *P. falciparum* between the *rpl*4 and *rpl*2 genes. Hypothetical protein genes were not found in Bsp/DH, *B. microti*, *T. parva*, *T. gondii* and *P. falciparum* between *rps*7 and *tuf*A, whereas BmLT/TZ, BmNX/HB, *B. bovis* and *B. orientalis* had two to seven hypothetical protein genes in the locus. Gene cluster 2 consisted of hypothetical protein genes and *Clp*C chaperone genes. Similar to *B. bovis*, *B. orientalis*, *B. microti* and *T. parva*, the *Clp*C genes of the six ovine *Babesia* isolates were duplicated, with both copies containing the AAA_2 ATPase domain, whereas *T. gondii* and *P. falciparum* contained one *Clp*C gene (Fig. 1).

Gene cluster 3 included *Rpo*B, *Rpo*C and *rps*2 genes, which were consistent with the gene orientation and contents of cluster 3 in *B. bovis*, *B. orientalis*, *B. microti* and *T. parva*. Cluster 3 of the piroplasma apicoplast genomes lacks the *suf*B gene involved in iron-sulphur cluster synthesis. Gene cluster 4 of the piroplasms had several tRNA genes and a single set of *SSU* and *LSU* genes, which are transcribed in the same orientation, whereas, this cluster consisted of two sets of *SSU* and *LSU* genes in opposite orientations in other parasites. Unlike BspXJ/DH, BmLT/TZ and BmNX/HB, one to two tRNA genes exist between the *SSU* and *LSU* genes in *B. bovis*, *B. orientalis*, *B. microti* and *T. parva* (Fig. 1).

### Comparison of genomic data sequenced by the Sanger dideoxy chain-termination method and Illumina technology

The apicoplast genomic sequences of BspXJ (KX881914) and BmLT (KX881915) sequenced using Illumina technology (designated BspXJ-Illumina and BmLT-Illumina) were compared with those obtained in this study using the Sanger dideoxy chain-termination method (designated BspXJ-Sanger, MH992224; BmLT-Sanger, MH992226). The results indicated that the size of the apicoplast genome, the number of tRNA and protein-encoding genes, and the initiation and termination codons used in the encoding genes are different in the two sets of data from Sanger and Illumina (Tables 1, 3). In addition, the A + T contents and the number of rRNA molecules were consistent but there were differences in the bases at several nucleotide positions along the full-length genome between the first-generation and second-generation data (Table 4).

**Table 4:** Comparison of *Babesia* sp. Xinjiang and *Babesia motasi* Lintan apicoplast genome were sequenced using Illumina technology (BspXJ-Illumina and BmLT-Illumina) and the Sanger dideoxy chain-termination method (BspXJ-Sanger and BmLT-Sanger)

Position^a^	5176-5249	5272	7495-7496	7503-7518	7524	7525-7532	7536-7538	9188	10128-10161	10168	10169	10172	16476	16783
*Babesia* sp. Xinjiang (BspXJ-Sanger, MH992224)		T	TT	AT…AG	A	GG…TA	GTA	G					T	C
*Babesia* sp. Xinjiang (BspXJ-Illumina, KX881914)		A	- -	-…-	C	-…-	- - -	C					G	T
*Babesia motasi* Lintan (BmLT-Sanger, MH992226)	TT…AA								CT…AA	A	G	G		
*Babesia motasi* Lintan (BmLT-Illumina, KX881915)	-…-								-…-	T	T	T		

^a^Position numbers given BspXJ (GenBank: MH992224)

*Key*: -, base deletion

### Phylogenetic analysis

**Fig. 2:**
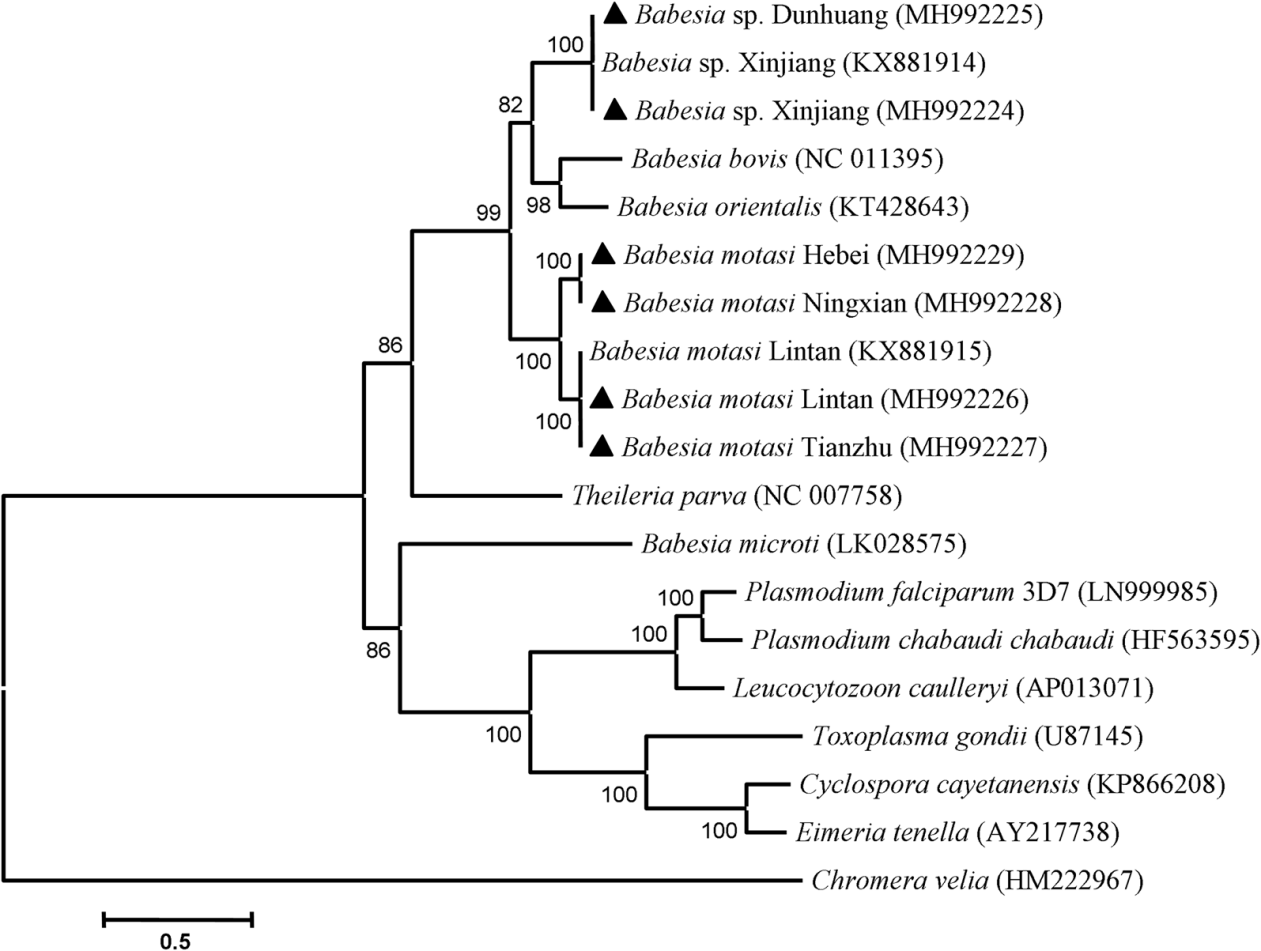
Phylogenetic relationships of *Babesia* infective to small ruminants in China and other apicomplexan parasites. Phylogeny was inferred with a maximum likelihood analysis of amino acid sequence data for 13 selected apicoplast genome-encoded genes based on distances calculated with the JTT with Freqs model. *Chromera velia* (HM222967) was used as the outgroup. Bootstrap values > 50% from 1000 replicates are shown on the nodes. *Babesia* obtained in this study is shown as triangles

The ML and NJ trees were constructed using the concatenated amino acid sequences of 13 gene products (4683 residues) from each apicoplast genomic sequence of 18 apicomplexan parasites and the chloroplast genomic sequence of *Chromera velie* (as an outgroup). The two approaches gave the same topology. In the amino acid phylogenetic trees, the piroplasms were divided into three groups: classical *Babesia* infective to ruminants, *Theileria* and *B. microti*. The classical *Babesia* species were separated into three clades: *B. motasi*, *Babesia* sp. Xinjiang/Dunhuang and *B. bovis*/*B. orientalis*. In addition, *B. motasi* were further divided into two subclades: *B. motasi* Lintan/Tianzhu and *B. motasi* Ningxian/Hebei (Fig. 2). In the whole apicoplast nucleotide phylogenetic tree, the grouping result for the six ovine *Babesia* isolates is the same as that of the amino acid phylogenetic trees (Additional file 10: Figure S8).

## Discussion

*Babesia* spp. are tick-transmitted haemoprotozoans that infect various animal species including humans, causing loss of livestock and public health concern in tropical and subtropical regions of southern Europe, Africa, Asia, Australia and the Americas [9, 36]. The apicoplast is a unique organelle found in apicomplexan parasites; it is considered a target for screening anti-parasitic drugs because it plays important roles in metabolic pathways for fatty acid, iron-sulphur, haem and isoprenoid biosynthesis [43]. In the present study, the apicoplast genomes of six ovine *Babesia* isolates were sequenced, assembled, annotated and compared with those of other apicomplexan parasites. The apicoplast genomes are smaller than those of most apicoplexan parasites [24, 28, 29, 33, 37, 44], but slightly larger than those of *P. chabaudi chabaudi* [35] and *B. microti* [25].

The content and order of genes in the cluster differ among the parasites. The *rpl*23, *rpl*11 and *suf*B genes are deficient in the apicoplast genomes of the six ovine *Babesia* spp., which is similar to *B. bovis*, *B. orientalis*, *B. microti*, *T. parva*, *T. gondii*, *E. tenella* and *C. cayetanensis*. It is possible that these genes were lost during the genetic evolution of the apicoplast in most apicomplexan parasites [25]. Therefore, some researchers speculate that the *rpl*23 and *rpl*11 genes do not play an important role in the growth and development of these parasites; alternatively, they may be translated at other sites in the apicoplast genome or directly encoded by the nuclear genome. The loss of the *suf*B gene may have been caused by the gene inversion of cluster 3 during the evolution of Piroplasmida [24, 25]. In this study, different numbers of hypothetical protein genes were found by aligning the apicomplexan apicoplast genomes, which suggests that *hyp* genes may be one of the causes of the variation in apicoplast genome size. Previous studies have suggested that rearrangements and loss of the genes involved in photosynthesis were thought to be responsible for the formation of apicoplast [24, 25]. Furthermore, gene deletion (*suf*B, *rpl*23, *rpl*11 and *hyp*), inversion (RNA polymerase), duplication (*Clp*C and *hyp*) and restructuring (*SSU* and *LSU*) were also important events during the early evolution of *Babesia* species. These modifications may have caused the apicoplast genomes of BmNX and BmHB to be the smallest among the reported *Babesia* in cattle and small ruminants.

Comparison of the BspXJ and BmLT apicoplast genome data sequenced by first-generation and second-generation sequencing techniques showed that there were some differences in genome sizes and the numbers of tRNA and protein-encoding genes. For example, *rpl*5 and *rpl*6 genes were present between *rpl*14 and *rps*5 genes in the second-generation sequence data of BmLT, while only the *rpl*6 gene exists in this region in the first-generation sequence data of the four *B. motasi* isolates. It is possible that these differences involving nucleotide substitutions, insertions and deletions were due to the different sequencing methods and annotation software used. Additionally, settings of a few parameters comprising ORF length, nested ORFs, start and stop codon, and genetic code were used. Therefore, it is recommended to perform first-generation sequencing to verify sequences based on the second-generation sequencing sequence, to annotate genes using two or more annotation software programs and then further improve the data by using BLAST.

Studies on the biological characteristics (including morphology, pathogenicity, vector tick, serology and *in vivo* or *in vitro* propagation) and molecular classification (target genes including *SSU*, ITS, *LSU*, *HSP90*, *cox*1, *cytb*, *cox*3, *RPS8* and *TRAP*) of the *Babesia* species infective to small ruminants in China have shown that these parasites are divided into two *Babesia* species: *B. motasi* and *Babesia* sp. There are two further subspecies of *B. motasi*, named BmLT/TZ and BmNX/HB (with differences in pathogenicity, serology, *in vitro* culture features and target gene sequences). These findings are consistent with the Uilenberg inference [12–15, 17, 18, 20, 38, 45–51]. In this study, phylogenetic analyses using concatenated amino acids or whole apicoplast nucleotide sequences confirmed the taxonomical relationships among *Babesia* species infective to small ruminants in China. In the ML and NJ trees, the ovine *Babesia* fell into two groups: *Babesia* sp. and *B. motasi*. *Babesia motasi* was further divided into two small clades, BmLT/TZ and BmHB/NX.

The apicoplast genomes in apicomplexan *Babesia* parasites remain conserved throughout all the species, including the six ovine *Babesia* isolates, and *Babesia* apicoplast functions are significantly different from those of the host [25], suggesting that they might be useful as targets for the development of potent and safe therapies for the treatment of babesiosis. Our data provide useful information confirming the taxonomical relationships of these parasites and identifying targets for anti-apicomplexan parasite drugs.

## Conclusions

In the present study, we have accomplished the sequencing, assembly and annotation of the apicoplast genomes of six ovine *Babesia* isolates from China. This study has also confirmed our previous inference that there are two *Babesia* species (*Babesia* sp. and *B. motasi*) infective for small ruminants in China, and that the four *B. motasi* isolates possibly belong to two subspecies (BmLT/BmTZ and BmNX/BmHB). Further studies are needed to validate the effects of anti-*Babesia* drugs against DNA replication, transcription, translation and 2-*C*-methyl-d-erythritol 4-phosphate pathways of the apicoplast.

## Additional files

**Additional file 1: Table S1.** Primers for amplifying apicoplast genomes of six ovine *Babesia* isolates.

**Additional file 2: Figure S1.** Circular map of the apicoplast genome of *Babesia* sp. Xinjiang.

**Additional file 3: Figure S2.** Circular map of the apicoplast genome of *Babesia* sp. Dunhuang.

**Additional file 4: Figure S3.** Circular map of the apicoplast genome of *Babesia motasi* Lintan.

**Additional file 5: Figure S4.** Circular map of the apicoplast genome of *Babesia motasi* Tianzhu.

**Additional file 6: Figure S5.** Circular map of the apicoplast genome of *Babesia motasi* Ningxian.

**Additional file 7: Figure S6.** Circular map of the apicoplast genome of *Babesia motasi* Hebei.

**Additional file 8: Table S2.** Comparison of BspXJ/BspDH, BmLT/BmTZ and BmNX/BmHB apicoplast genomes.

**Additional file 9: Figure S7.** Whole genome alignment of *Babesia* obtained in this study with ten genomes from apicomplexan parasites: *B. bovis*, *B. orientalis*, *B. microti*, *C. cayetanensis*, *E. tenella*, *L. caulleryi*, *P. chabaudi chabaudi*, *P. falciparum*, *T. parva* and *T. gondii*. Comparison was performed using Mauve. The coloured blocks in the first genome are connected by lines to similar blocks in the other genomes. The region of sequence covered by a coloured block is entirely collinear and homologous among the genomes. Each locally collinear block (LCB) is assigned a unique colour and the apicoplast assemblies presented re-arrangements in some species.

**Additional file 10: Figure S8.** Phylogenetic relationships of six ovine *Babeisa* isolates and other apicomplexan parasites. Phylogeny was inferred with a maximum likelihood analysis of whole nucleotide sequences based on distances calculated with the Kimura 2-parameter model. *Chromera velia* (HM222967) was used as the outgroup. Bootstrap values > 50% from 1000 replicates are shown on the nodes. *Babesia* obtained in this study is shown as triangles.

## Data Availability

The datasets generated or analyzed during this study are included in this published article and its additional files. The newly generated sequences were submitted to the GenBank database under the accession numbers MH992224-MH992229.

## Abbreviations

DNA: deoxyribonucleic acid
PCR: polymerase chain reaction
ORF: open reading frame
tRNA: transfer ribonucleic acid
rRNA: ribosomal RNA
*SSU*: small subunit ribosomal RNA
*LSU*: large subunit ribosomal RNA
*rpl*: ribosomal protein large subunits gene
*rps*: ribosomal protein small subunits gene
*tufA*: elongation factor TU gene
*ropB*: DNA-directed RNA polymerase subunit beta gene
*rpoC*: DNA-directed RNA polymerase subunit gene
*ClpC*: ATP-dependent *Clp* protease gene
hyp: hypothetical protein
*ITS*: internal transcribed spacer gene
*HSP90*: heat-shock protein 90 gene
*cox*1: cytochrome *c* oxidase subunit 1 gene
*cytb*: cytochrome b gene
*cox*3: cytochrome *c* oxidase subunit 3 gene
*RPS8*: ribosomal protein S8 gene
*RAP-1*: rhoptry-associated-protein-1 gene
*TRAP*: thrombospondin-related anonymous protein gene
Fe:S: iron-sulphur
FASII: type II fatty-acid biosynthesis
MEP: 2-*C*-methyl-d-erythritol 4-phosphate
