# Supplementary figures and images for: Comparative analysis of apicoplast genomes of Babesia infective to small ruminants in China
Source: Parasit Vectors. 2019 Jun 24;12:312. doi: 10.1186/s13071-019-3581-x (PMC6591869; doi:10.1186/s13071-019-3581-x)

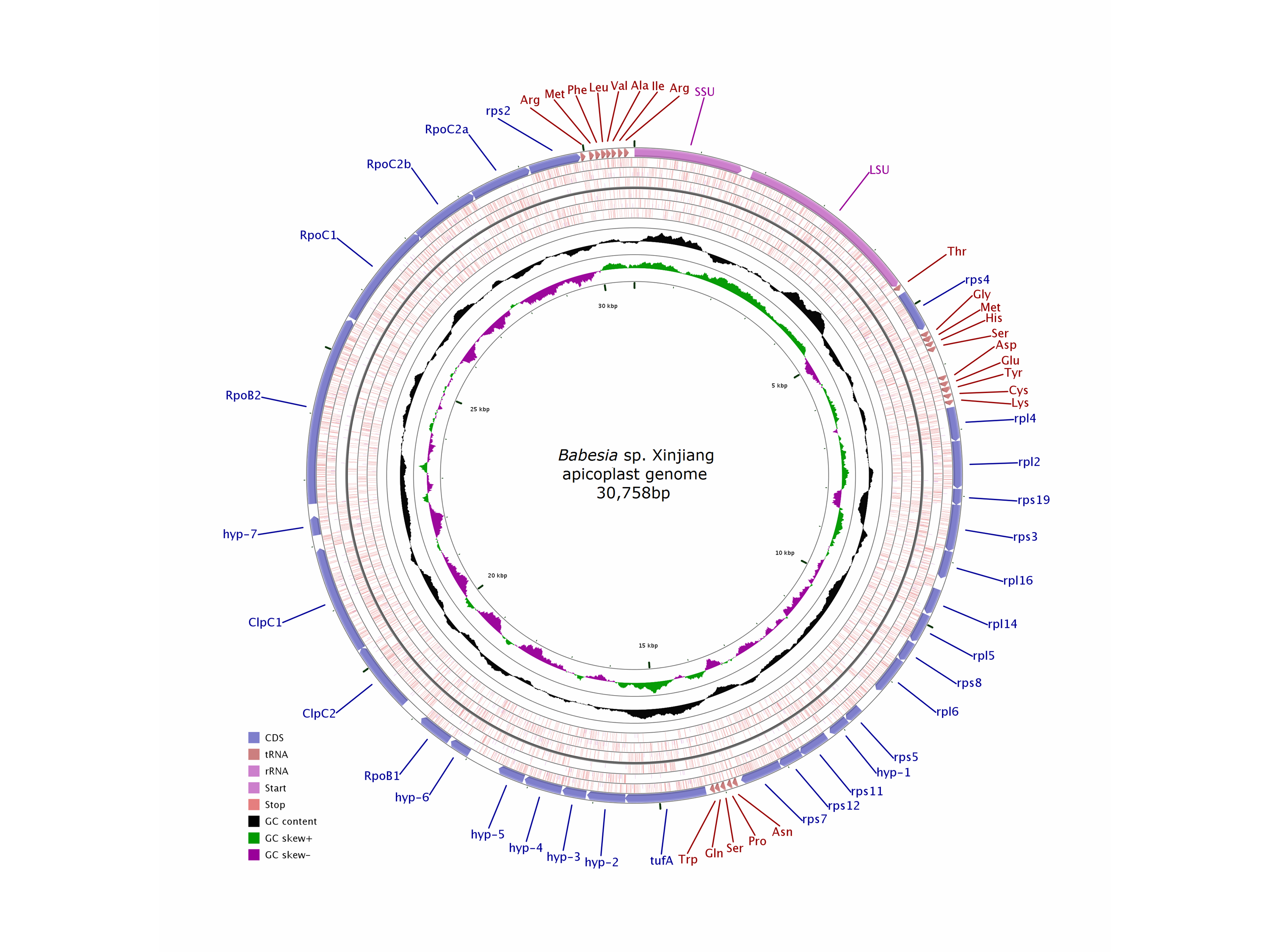

Supplement: Supplementary file 2 — Additional file 2: Figure S1. Circular map of the apicoplast genome of Babesia sp. Xinjiang. [file 13071_2019_3581_MOESM2_ESM.tif]

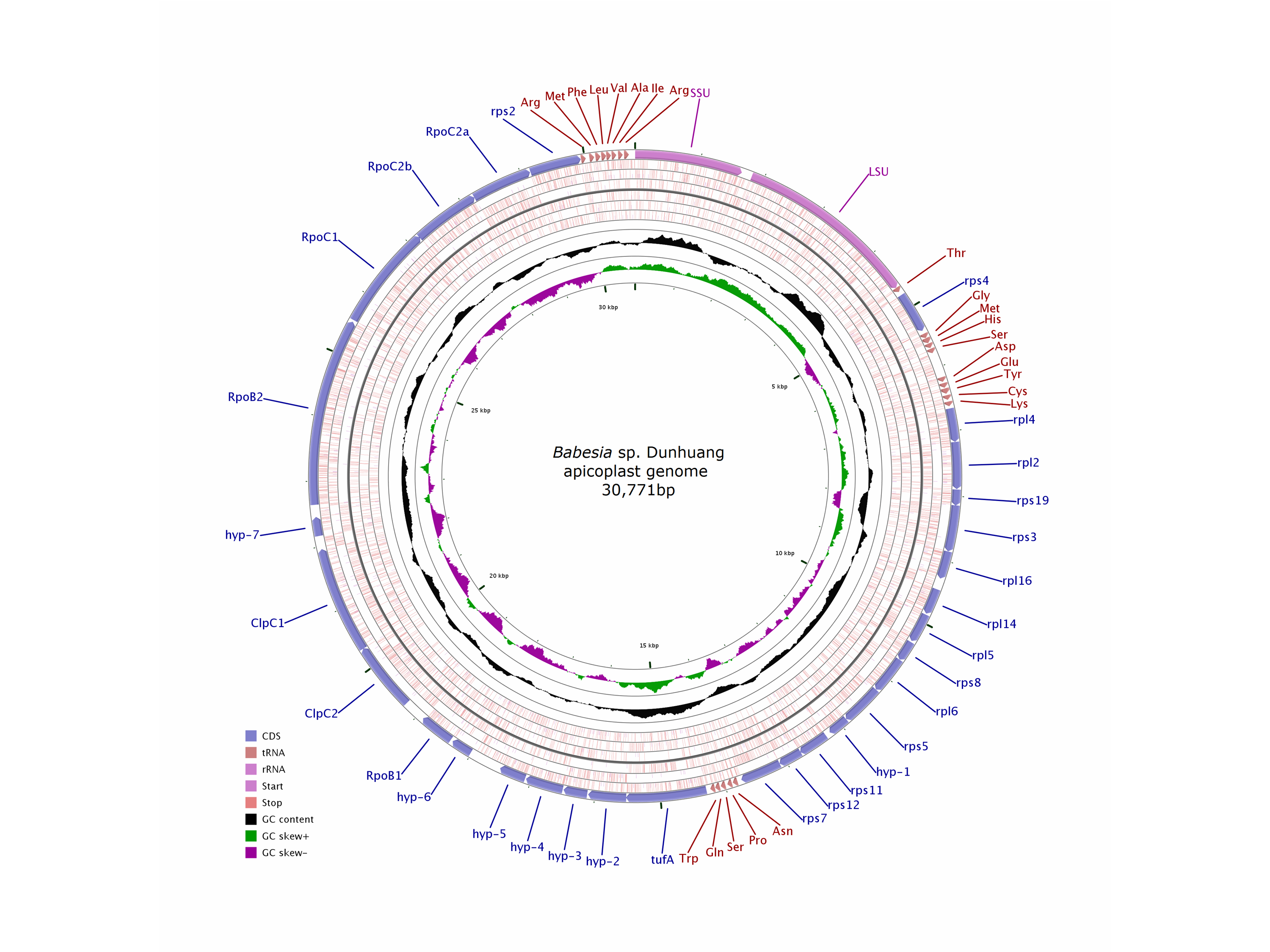

Supplement: Supplementary file 3 — Additional file 3: Figure S2. Circular map of the apicoplast genome of Babesia sp. Dunhuang. [file 13071_2019_3581_MOESM3_ESM.tif]

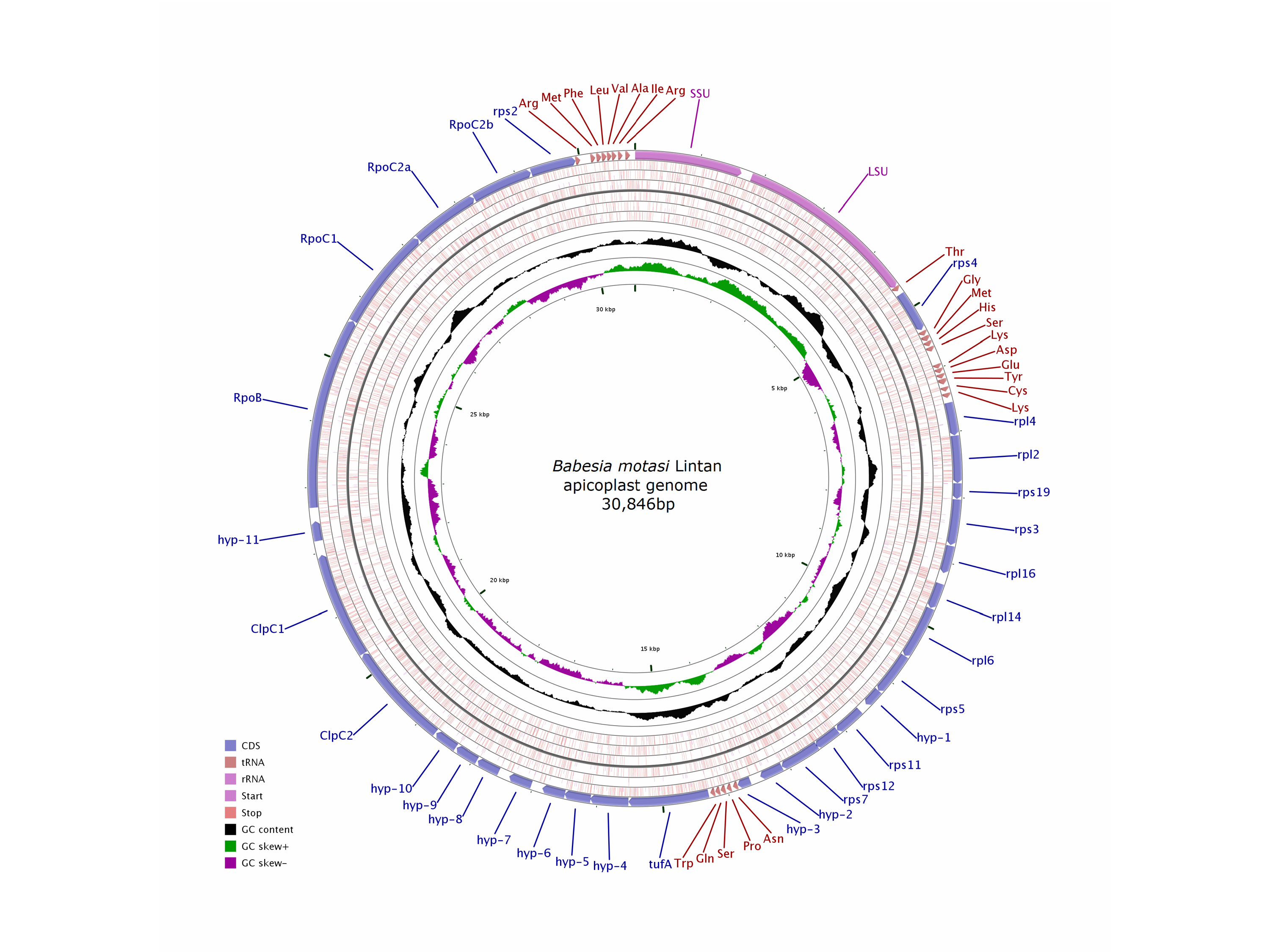

Supplement: Supplementary file 4 — Additional file 4: Figure S3. Circular map of the apicoplast genome of Babesia motasi Lintan. [file 13071_2019_3581_MOESM4_ESM.tif]

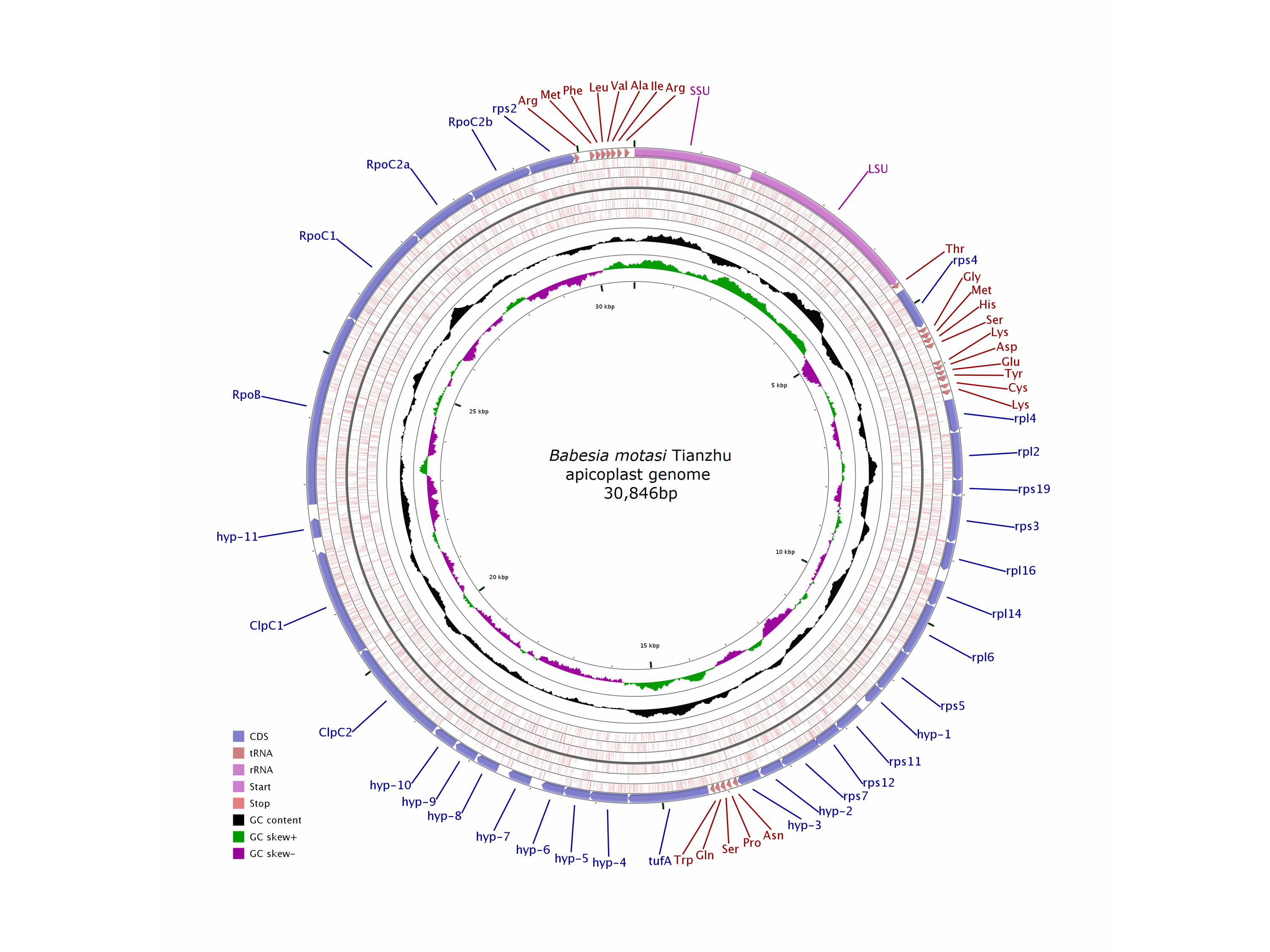

Supplement: Supplementary file 5 — Additional file 5: Figure S4. Circular map of the apicoplast genome of Babesia motasi Tianzhu. [file 13071_2019_3581_MOESM5_ESM.tif]

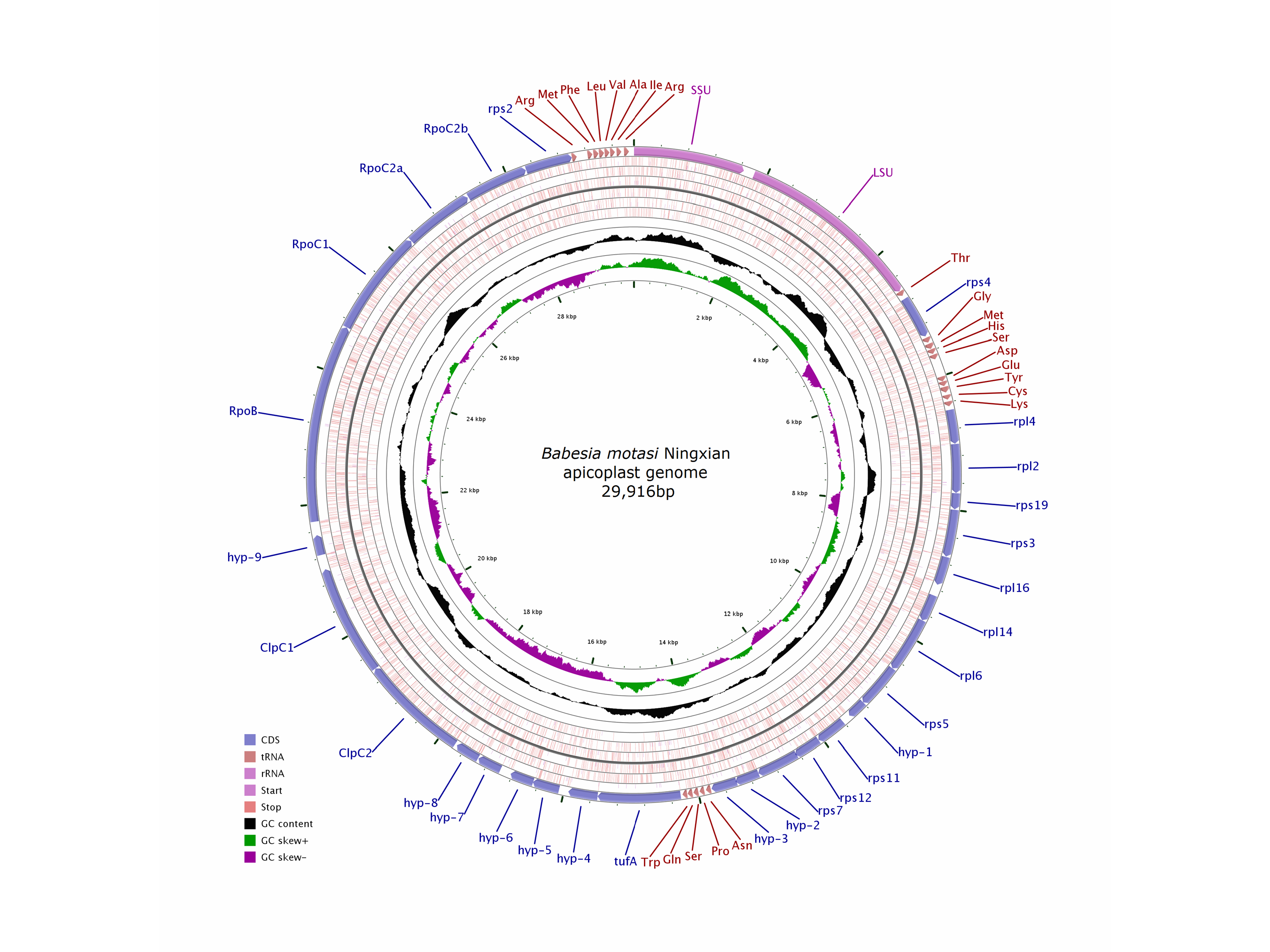

Supplement: Supplementary file 6 — Additional file 6: Figure S5. Circular map of the apicoplast genome of Babesia motasi Ningxian. [file 13071_2019_3581_MOESM6_ESM.tif]

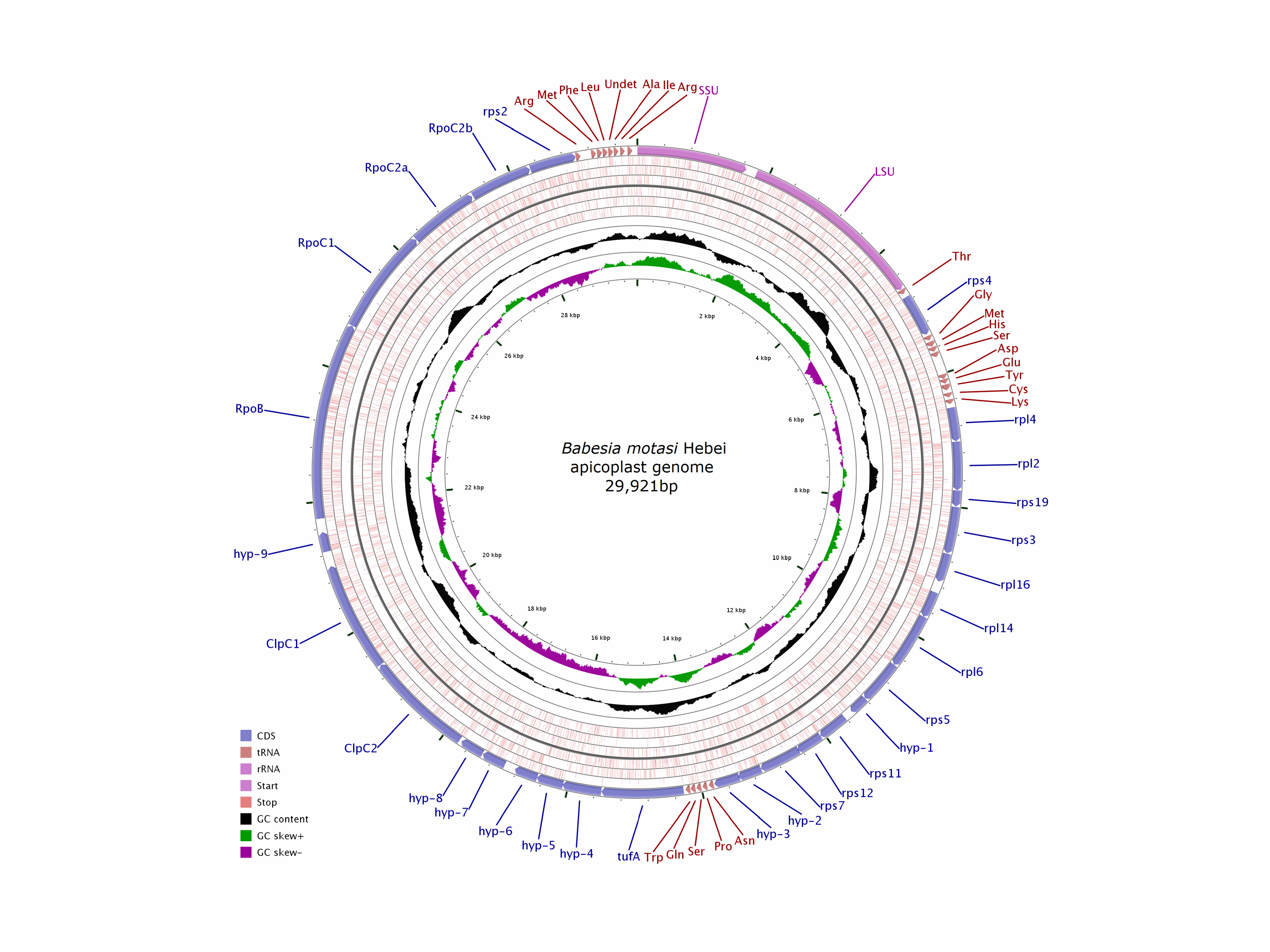

Supplement: Supplementary file 7 — Additional file 7: Figure S6. Circular map of the apicoplast genome of Babesia motasi Hebei. [file 13071_2019_3581_MOESM7_ESM.tif]

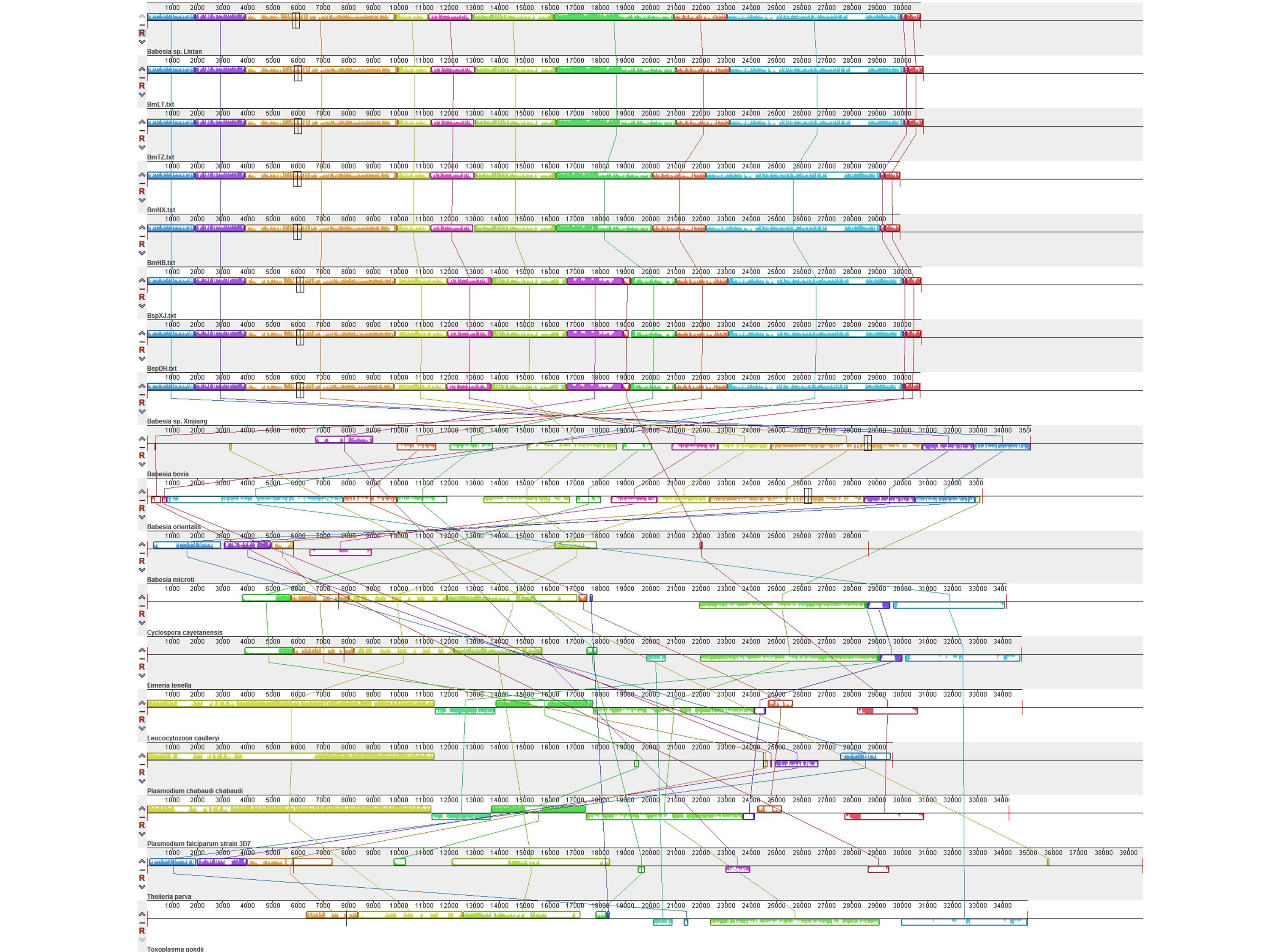

Supplement: Supplementary file 9 — Additional file 9: Figure S7. Whole genome alignment of Babesia obtained in this study with ten genomes from apicomplexan parasites: B. bovis, B. orientalis, B. microti, C. cayetanensis, E. tenella, L. caulleryi, P. chabaudi chabaudi, P. falciparum, T. parva and T. gondii. Comparison was performed using Mauve. The coloured blocks in the first genome are connected by lines to similar blocks in the other genomes. The region of sequence covered by a coloured block is entirely collinear and homologous among the genomes. Each locally collinear block (LCB) is assigned a unique colour and the apicoplast assemblies presented re-arrangements in some species. [file 13071_2019_3581_MOESM9_ESM.tif]

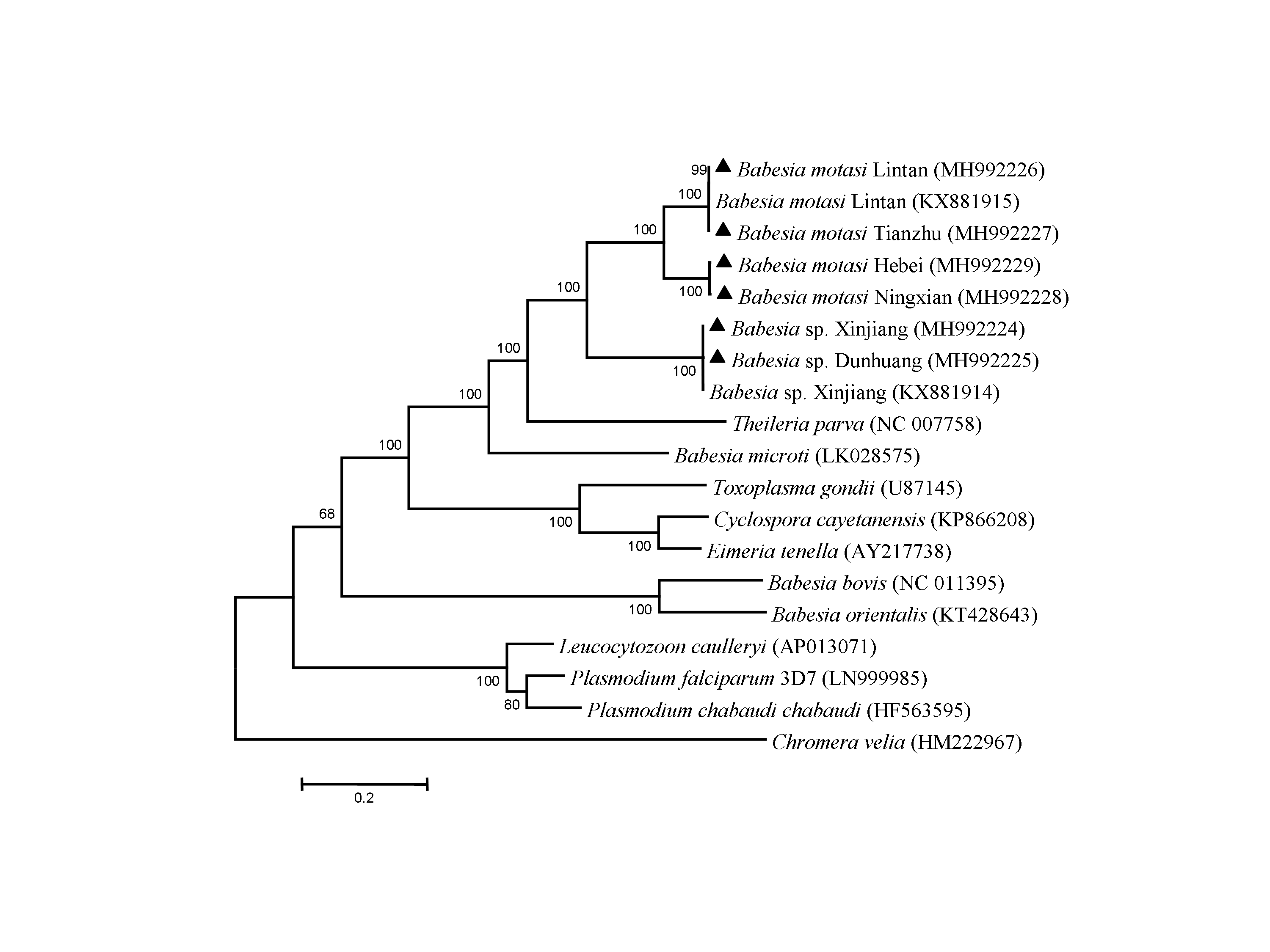

Supplement: Supplementary file 10 — Additional file 10: Figure S8. Phylogenetic relationships of six ovine Babeisa isolates and other apicomplexan parasites. Phylogeny was inferred with a maximum likelihood analysis of whole nucleotide sequences based on distances calculated with the Kimura 2-parameter model. Chromera velia (HM222967) was used as the outgroup. Bootstrap values > 50% from 1000 replicates are shown on the nodes. Babesia obtained in this study is shown as triangles. [file 13071_2019_3581_MOESM10_ESM.tif]
